# Overrepresented and Underserved? Autism in Crisis Team Referrals

**DOI:** 10.1192/bjo.2026.11419

**Published:** 2026-06-30

**Authors:** Jun Jie Lim, Adam Richards, Chloe Fallon-Smith, Gaelle Slater

**Affiliations:** 1 School of Medicine and Population Health, University of Sheffield, Sheffield, United Kingdom; 2 Crisis Resolution and Home Treatment Team, Sheffield Health and Social Care NHS Foundation Trust, Sheffield, United Kingdom

## Abstract

**Aims:**

Autistic adults are at increased risk of mental health crises, yet their representation within crisis services and staff preparedness remain under-researched. This study aimed to determine the prevalence of diagnosed and suspected autism spectrum disorder (ASD) among referrals to a Crisis Resolution and Home Treatment Team (CRHTT), describe associated crisis presentations, and assess staff training experiences and knowledge.

**Methods:**

A retrospective observational service evaluation was conducted within a crisis team in Sheffield, UK. A random sample of 400 adult referrals was selected from 2397 referrals. Demographic data, ASD diagnostic status, and presenting complaints were extracted from electronic records. Staff knowledge and training were assessed using an adapted National Autism Implementation Team questionnaire. Quantitative data were analysed descriptively and compared with population prevalence estimates; qualitative free-text responses were analysed thematically.

**Results:**

Among the 400 referrals, 10.5% had a formal ASD diagnosis and 11.3% were suspected cases, yielding a combined prevalence of 21.8%, over nine times higher than general population estimates. Common crisis presentations included suicidal thoughts or behaviours (41%), acute psychotic episodes (24%), and worsening psychiatric comorbidities (18%). One-third of suspected ASD cases had not been referred for diagnostic assessment. Forty percent of staff reported no ASD-specific training, and only 10% rated their knowledge as full. Qualitative findings highlighted insufficient, overly generic training and low confidence in managing autistic adults in crisis.

**Conclusion:**

Autistic adults are substantially overrepresented in mental health crisis services, yet diagnostic delays and limited staff training persist. Improving autism-specific training, strengthening diagnostic referral pathways, and adopting more proactive models of care are essential to enhance crisis support and reduce repeated emergency presentations among autistic adults.

